# Automated retinal boundary segmentation of optical coherence tomography images using an improved Canny operator

**DOI:** 10.1038/s41598-022-05550-y

**Published:** 2022-01-26

**Authors:** Jian Liu, Shixin Yan, Nan Lu, Dongni Yang, Hongyu Lv, Shuanglian Wang, Xin Zhu, Yuqian Zhao, Yi Wang, Zhenhe Ma, Yao Yu

**Affiliations:** 1 School of Control Engineering, Northeastern University at Qinhuangdao, Qinhuangdao, 066004 China; 2 Hebei Key Laboratory of Micro-Nano Precision Optical Sensing and Measurement Technology, Qinhuangdao, 066004 China; 3 Department of Ophthalmology, The First Hospital of Qinhuangdao, Qinhuangdao, 066004 China; 4 Department of Ophthalmology, Qinhuangdao Maternal and Child Health Hospital, Qinhuangdao, 066004 China; 5 Tangshan Maternal and Children Hospital, Tang shan, 063000 China; 6 Biomedical Information Engineering Lab, The University of Aizu, Aizu-Wakamatsu, Fukushima 9650053 Japan

**Keywords:** Biomedical engineering, Image processing

## Abstract

Retinal segmentation is a prerequisite for quantifying retinal structural features and diagnosing related ophthalmic diseases. Canny operator is recognized as the best boundary detection operator so far, and is often used to obtain the initial boundary of the retina in retinal segmentation. However, the traditional Canny operator is susceptible to vascular shadows, vitreous artifacts, or noise interference in retinal segmentation, causing serious misdetection or missed detection. This paper proposed an improved Canny operator for automatic segmentation of retinal boundaries. The improved algorithm solves the problems of the traditional Canny operator by adding a multi-point boundary search step on the basis of the original method, and adjusts the convolution kernel. The algorithm was used to segment the retinal images of healthy subjects and age-related macular degeneration (AMD) patients; eleven retinal boundaries were identified and compared with the results of manual segmentation by the ophthalmologists. The average difference between the automatic and manual methods is: 2–6 microns (1–2 pixels) for healthy subjects and 3–10 microns (1–3 pixels) for AMD patients. Qualitative method is also used to verify the accuracy and stability of the algorithm. The percentage of “perfect segmentation” and “good segmentation” is 98% in healthy subjects and 94% in AMD patients. This algorithm can be used alone or in combination with other methods as an initial boundary detection algorithm. It is easy to understand and improve, and may become a useful tool for analyzing and diagnosing eye diseases.

## Introduction

The structural features of retina have been shown to be closely related to many ophthalmological diseases. For example, the thickness of the retina, especially the nerve fiber layer (NFL), has been used to indicate the progression of glaucoma^1^. It has been reported that the junction of the Inner segment (IS) and the Outer segment junction (OSJ) can facilitate the diagnosis of retinitis pigmentosa^2,3^. Quantitative assessment of retinal pigment epithelium (RPE) is also useful in diagnosing some age-related macular degeneration (AMD)^4^. Thus, quantitative research on the retinal features has clinical value. Optical coherence tomography (OCT), as a noninvasive, label-free and high-resolution imaging modality, has been proven useful to diagnose various retinal diseases^5^. The accurate and reliable segmentation of retinal layers in OCT images is a key step in the quantitative study of retinal features.

Commercial OCT devices are generally equipped with some kind of image analysis software which is able to perform retinal segmentation with varying success rates^6^. However, the details of their design are undisclosed^7^. In addition, most methods are designed for specific equipment^8^. This makes them difficult to replicate in independent studies or to be improved by other scholars. Machine learning approaches, including support vector machines^9^, random forest^10^, and Bayesian artificial neural networks^11^, are proven to obtain satisfactory solutions in a noisy environment^12^. However, the huge amount of labeled data used in machine learning is difficult to obtain. Relying solely on manual segmentation will make this task extremely difficult. Furthermore, manual segmentation is greatly affected by changes in observers^13^. Therefore, the automatic retina segmentation algorithm based on non-machine learning still has certain practical value. It can be used alone, or it can provide a large amount of label data for machine learning under human supervision.

Anisotropic filtering seems very promising for improving contrast and boundary detection rate^14^, but it also enhances some vertical boundary caused by vascular shadows. In order to avoid this influence, neighborhood information is often used by some algorithms. The prerequisite is that the retinal layers tend to be horizontal. So, A-lines alignment on the OCT images is often required before layer segmentation. Qi Yang et al.^15^ used a large size gradient template to incorporate adjacent information and deal with vascular shadow and artifacts. Before that, the IS/OS boundary was first detected, and then the images were further aligned along the IS/OS boundary. Garvin et al. proposed a 3D graph search approach, which need to first align all the slices and straightened the RPE layer^16^. Zhang et al. proposed a segmentation algorithm based on two-step predenoising filtering, which can only be executed after A-lines alignment^17^. However, a number of severe eye diseases (AMD, choroidal neovascularisation (CNV), glaucoma, etc.) can change the structure of retina and choroid^18^. In this case, A-lines alignment cannot be performed. The active contour model is good at finding local optima, but its limitation is that the algorithm cannot converge to the optimal boundary unless the initial point is close enough^19,20^. Additionally, the active contour model as a semi-automatic segmentation algorithm requires too much human involvement. Apparently, if an algorithm relies on human involvement heavily, it will be difficult to deal with large amounts of data, and more importantly, its performance is difficult to evaluate objectively. Therefore, the development of a retinal segmentation algorithm with high accuracy and robustness without human intervention has important clinical significance.

Among the fully automatic edge detection algorithms, Canny operator is recognized as the best operator so far and is often used to obtain the initial boundary of the retina in retinal segmentation. Unfortunately, if the Canny operator is applied to retinal edge detection without any modification, it will face some limitations. First, it is susceptible to the shadow of blood vessels and detects many borders perpendicular to the retinal layer. Second, the detected boundary may be discontinuous, especially when there are many blood vessels or the image quality is not ideal. Additionally, it is sensitive to noise interference and is prone to misdetection or missed detection. Qi Yang et al. combined Canny edge maps and the axial intensity gradient maps to segment the retinal layer^15^. The axial intensity gradient maps provide complementary search guidance where Canny edge information is missing or weak. The layer boundary is then extracted by a shortest path search applied to the graph using a dynamic programming algorithm. Although this method can alleviate part of the problem of edge discontinuity, the vertical boundary caused by blood vessel shadow can only be weakened, but cannot be completely eliminated, which increases the uncertainty of boundary selection.

In this paper, we proposed an automated retinal layer segmentation algorithm based on improved Canny operator. This method adds a multi-point boundary search step on the basis of the traditional Canny operator and adjusts the convolution kernel function. The improved Canny operator has a dramatic improvement in the extraction of the retinal boundary. To make it easier to understand, we divide the algorithm into three parts: First, Image enhancement, this section includes image denoising, gradient calculation and non-maximum suppression, which are similar to but slightly different from the first three steps of Canny operator. Second, boundary search, multiple gradient peak points are selected as seed points to search the retinal boundaries and superimpose the obtained boundaries together. The third, Boundary selection, the number of superposed boundaries is converted into probabilities, and the double threshold method in Canny operator is used to select and connect the edges. This method can accurately distinguish eleven retinal boundaries without additional intervention such as A-lines alignment, manual initialization, parameter adjustment or search space restriction. Quantitative and qualitative methods were used to verify the accuracy and stability of the algorithm. We also compared the proposed method with a state-of-the-art method using public healthy and AMD eye data sets.

## Related work

Various automatic retinal segmentation algorithms have been proposed by previous reports. These algorithms can be divided into two main categories: data-driven approaches^21–24^ and algorithm-driven approaches^25–28^. The former mainly refers to methods based on traditional machine learning and deep learning. These methods train classifiers using large amounts of data set to assign specific categories to each pixel in the retinal images. For example, support vector machine^9,29,30^, random forest^10^ and convolutional neural network^21,22,31–34^. Algorithm-driven approaches, such as level set, graph theory and dynamic programming, mainly construct mathematical model of retinal boundary segmentation by capturing the anatomical information and optical characteristics of the retinal layer.

Novosel et al., developed a loosely coupled level set method to segment retinal layers coupling based on the order of layers and thickness priors but only eight interfaces were detected in the OCT images from normal retinas^35^. Subsequently, they use the same method to segment the diseased retinal layers, but only seven surfaces were detected^36^.Wang et al.,^37^ utilized a fuzzy level set-based method to segment diabetic macular edema images.

Compared with other mathematical models, the graph theory based methods have obvious advantages both in algorithm performance and algorithm complexity^38^. Chiu et al.^13^ proposed a Graph theory and Dynamic Programming (GTDP) approach for automatic segmentation of seven retinal layers. Two years later, they extended the GTDP algorithm to the segmentation of retina layer with AMD, taking into account the clinical characteristics of the disease on the basis of the original algorithm, and successfully segmented three boundaries^39^. Garvin et al.^40^ optimized the cost function and proposed a method that can be directly used for retinal layer boundary segmentation in 3D OCT images. Xiang et al.^41^ proposed a multi-resolution graph search method to perform simultaneous layer segmentation and fluid segmentation. Recently, Hussain et al.^42^ proposed a novel approach to construct graph models. They first extract candidate boundary pixel groups using Canny edge detection operator, and then regarded their endpoints as the nodes of the graph. In 2017, LD Sisternes et al.^43^ proposed a novel segmentation method based on iterative adaptation of a weighted median process, in which a three-dimensional weighting function is defined considering image intensity and gradient properties.

Dynamic programming algorithms, due to their significant advantages in solving optimization strategy problems, have been widely used in retina segmentation. Koozekanani et al. used a Markov boundary model to connect the rough edge^44^. But it is sensitive to noise, so the detected layer boundaries can easily deviate from the true boundary. Mishra et al.^20^ presented a promising two-step algorithm based on a kernel optimization scheme. First, approximate positions of the boundaries were found, and then by using dynamic programming the boundaries were refined to obtain the ideal segmentation results; however, no quantitative evaluation on a large data set was given. Stephanie J. et al. proposed an automatic initialization method that bypasses the need for manual endpoint selection of the dynamic programming algorithm^13^. Tian et al.^45^ proposed a shortest path based graph search method to detect retinal boundaries by searching the shortest path between nodes at both ends. The time complexity was reduced by the limitation of the search region and down-sampling. In order to prevent the algorithm from accidentally segmenting other structures in place of the target feature, it is often necessary to limit the graph to a valid search space that excludes any irrelevant content when using dynamic programming methods. However, the selection of search space will be adjusted artificially according to actual needs.

## Materials and methods

### Characteristics and nomenclature of retinal layer

**Figure 1:**
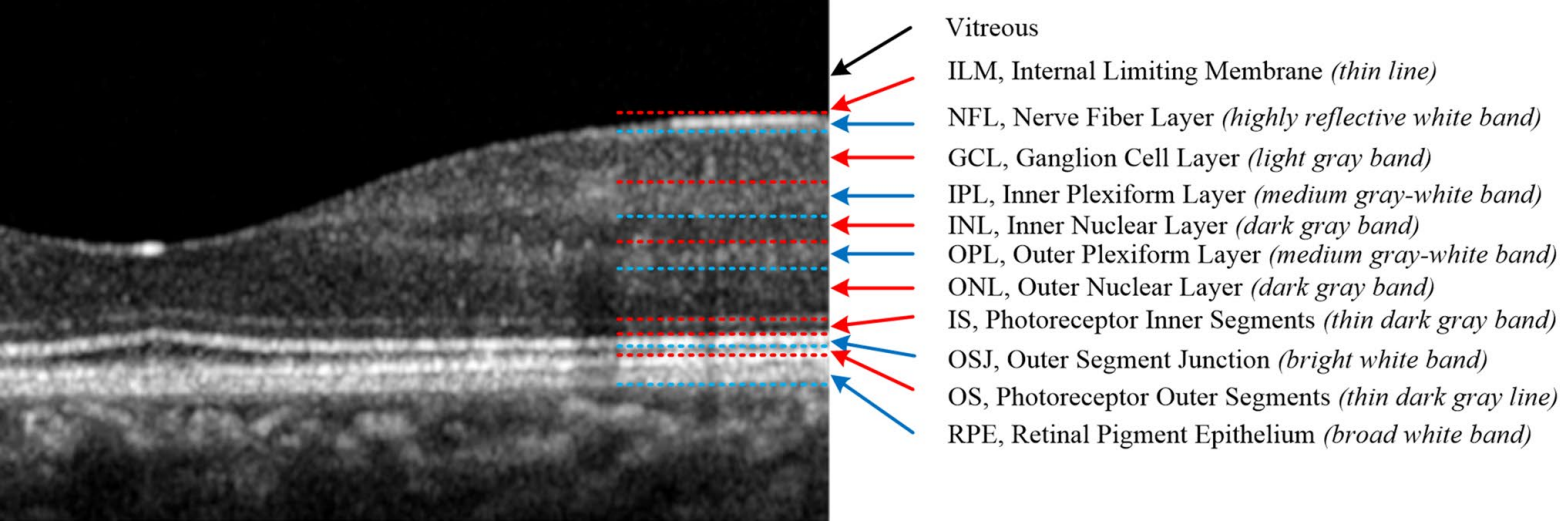
Illustration of the position of eleven retinal layers. The figure also indicates the full names, abbreviations and the optical characteristics of each layer.

Figure 1 shows a cross sectional OCT image of a normal retina centered at the macula. The positions, full names and abbreviations of the eleven retinal layers are marked, and the light and dark characteristics of each retinal layer are also indicated.

Each retinal layer is a biological tissue with a specific thickness; however, this paper aims to identify each retinal boundary. For convenience, we adopted Luis’s^43^ naming method. The bottom boundary of each retinal layer was named with the prefix "o-" plus the abbreviation of the retinal layer. For example, the boundary between NFL and GCL, we call it o-NFL; the boundary between OPL and ONL, we call it o-OPL, and so on. ILM is a thin layer, so it is treated as a boundary directly. Among the eleven boundaries in the retinal image, the ones from dark to bright include: ILM, o-GCL, o-INL, o-ONL, o-IS and o-OS, as shown in Fig. 1, indicated by red arrows; the borders from bright to dark includes: o-NFL, o-IPL, o-OPL, o-OSJ and o-RPE, indicated by blue arrows.

### Improved Canny algorithm

**Figure 2:**
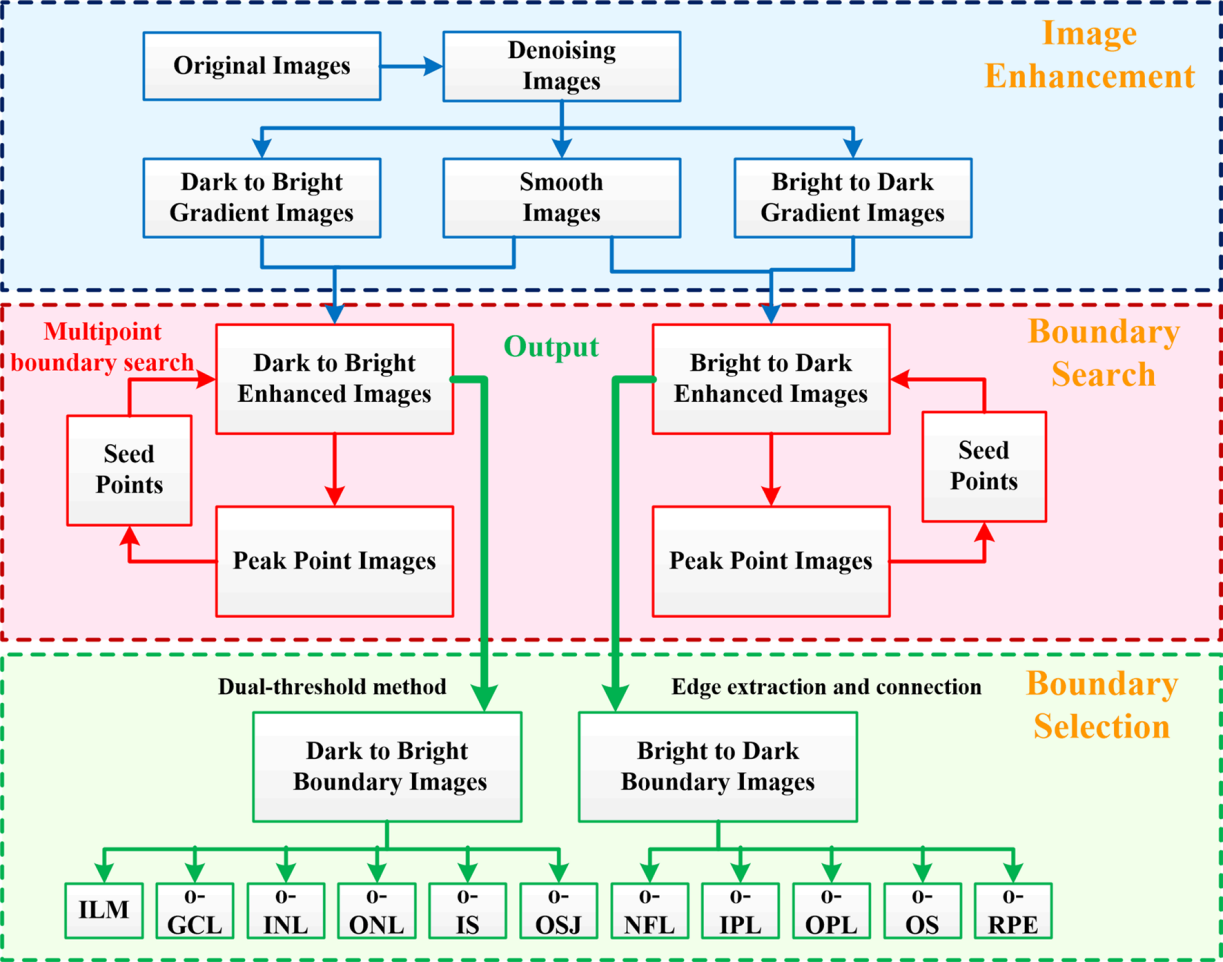
Schematic of the segmentation steps.

The algorithm in this paper is divided into three parts: (1) Image enhancement, (2) Boundary search, (3) Boundary selection. The schematic of the segmentation steps is shown in Fig. 2.

(1) Image enhancement

**Figure 3:**
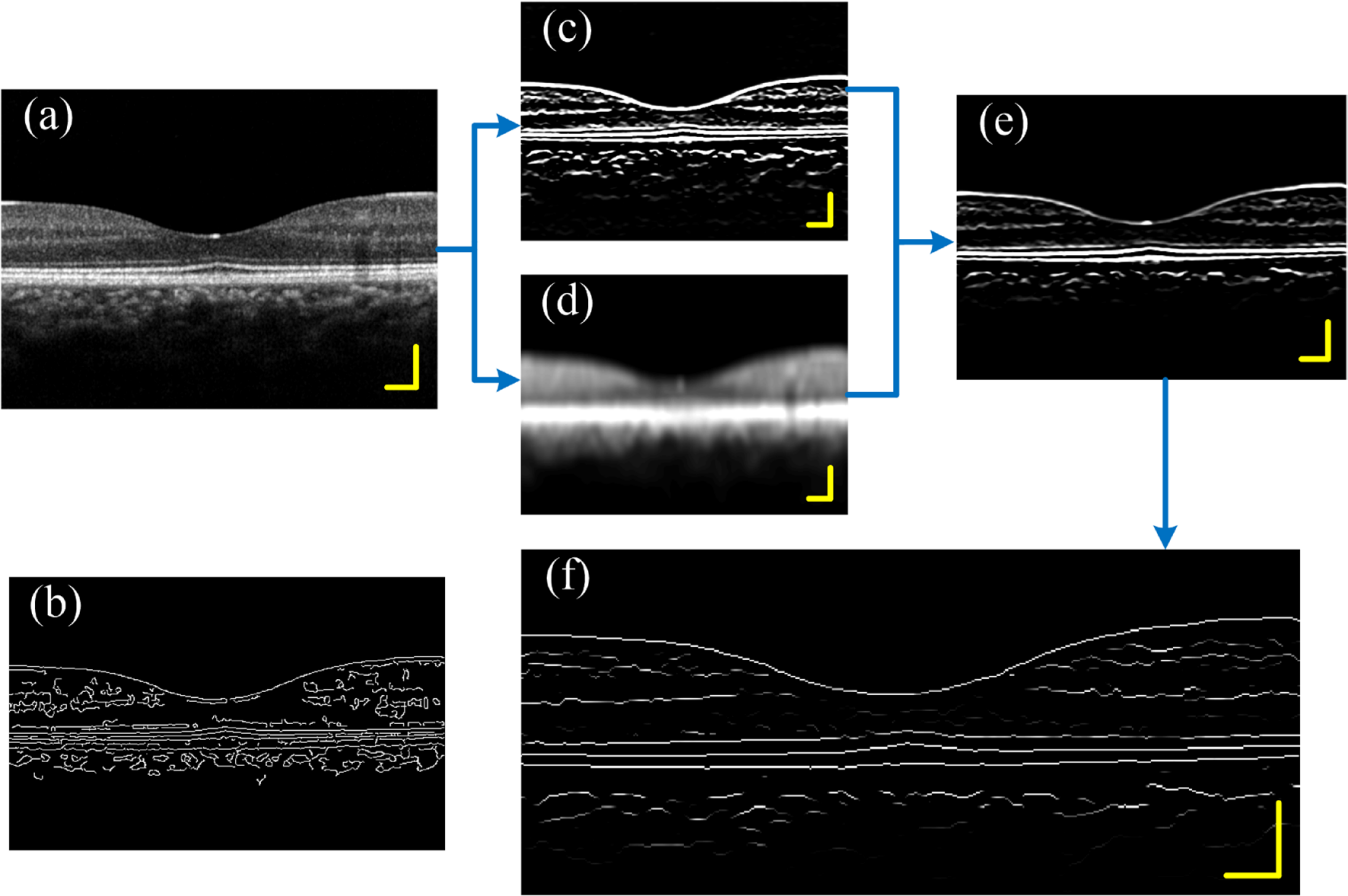
Image edge enhancement. (**a**) Image after denoising. (**b**) Boundary detection result obtained by using traditional canny operator. (**c**) Gradient image. (**d**) Large-scale smooth image. (**e**) Boundary enhanced image. (**f**) Peak point image. Scale bar length is 200 μm.

This section covers image denoising, gradient calculation, and non-maximum suppression, which are similar to the first three steps of the Canny operator. First, a 3*3 Gaussian filter template is used for image denoising, and the filtered image is shown in Fig. 3a. Figure 3b is the boundary detection result obtained by using traditional canny operator. It can be seen that the detected boundaries are discontinuous and contain a large number of axial boundaries. In order to mainly highlight the horizontal edge, an axial gradient template is used on the gradient calculation part. The retinal image contains two types of boundaries, from dark to bright and from bright to dark. The two types of boundaries are enhanced using the ascending gradient template (4 × 1 template, as show in Eq. 1) and the descending gradient template respectively (4 × 1 template, as show in Eq. 2).

1 $$ \left[ { - 1,{ } - 1,{ }1,{ }1{ }} \right]^{{\text{T}}} $$

2 $$ \left[ {1,{ }1,{ } - 1,{ } - 1{ }} \right]^{{\text{T}}} $$

The 4 × 1 gradient template is more robust than the 2 × 1 gradient template. The gradient image (Fig. 3c) is obtained by convolving the filtered image with the gradient template (Eq. 1). On the other hand, we perform large-scale smoothing on Fig. 3a to obtain smooth image (Fig. 3d), the size of the smoothing template is^9,10^. The boundary enhancement image (Fig. 3e) is obtained by multiplying the gradient image (Fig. 3c) and the smooth image (Fig. 3d) point-to-point. The purpose of this step is to weaken the interference outside the retinal image, such as posterior vitreous face or image noise. Then, the "non-maximum suppression" method is performed along the A-scan direction to obtain the peak point images (Fig. 3f). These peak points constitute the initial boundary of the image, but some of these boundaries are discontinuous. Thus, on this basis, we added a multi-point boundary search method to improve the image boundary. The length of the scale bar in all the pictures in this article is 200 μm.

(2) Boundary search

**Figure 4:**
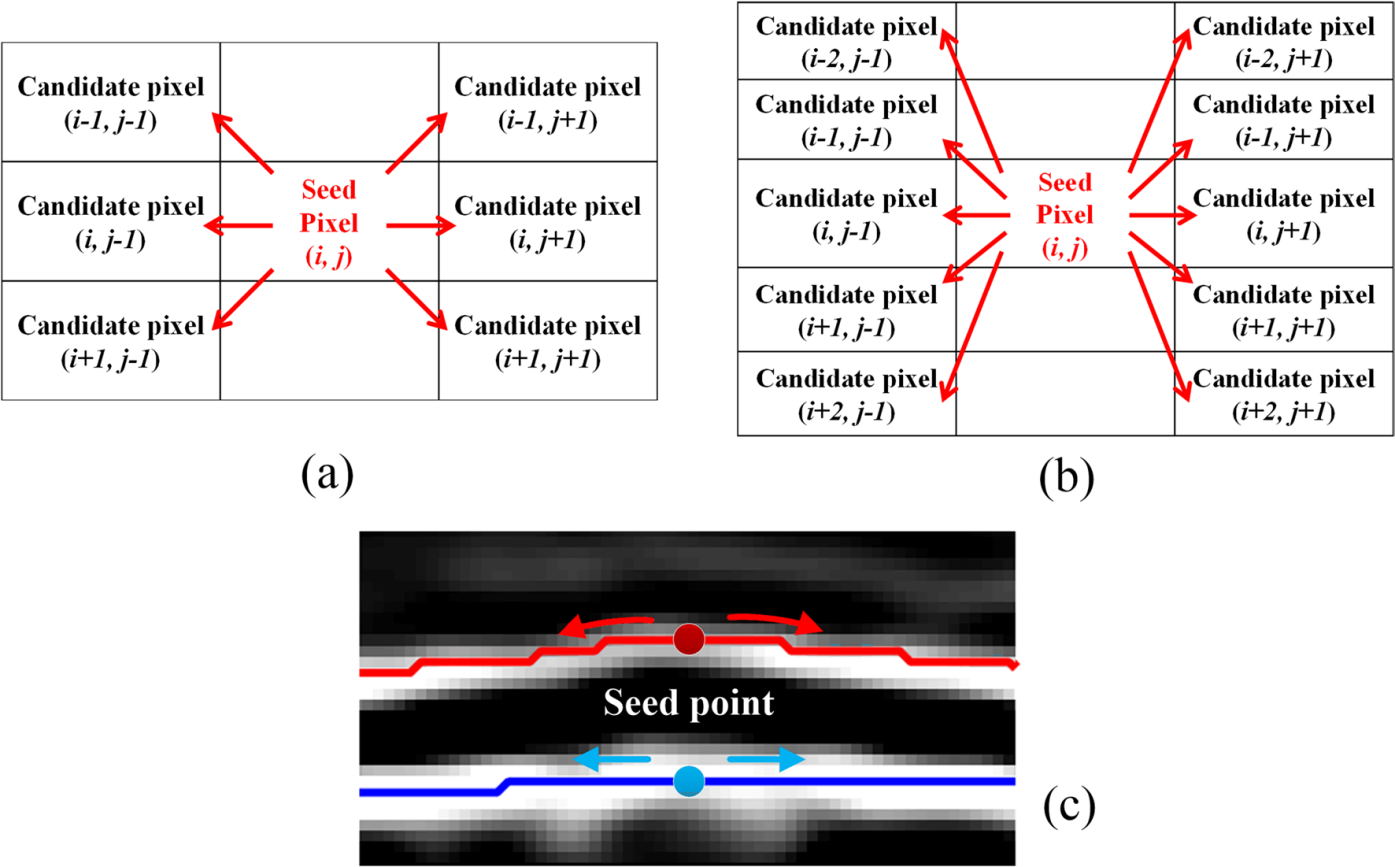
Three neighborhoods (**a**) and five neighborhoods (**b**) boundary search criterion. (**c**) Boundary search diagram. The red and blue solid circles represent seed points on different boundaries, and the arrow indicates the search direction.

Most of the points in Fig. 3f are located at or close to the boundary of the retina. So, these points are used as seed points to search for retinal boundaries in the gradient image (Fig. 3e). The search criterion is to select the neighboring point closest to the seed point's intensity as the potential boundary pixels, and then use this point as a new seed point to repeat the previous process until it extends to the first or last column of the image. The search direction can be 3-neighborhoods or 5-neighborhoods, as shown in Fig. 4a,b. 3-neighborhoods are suitable for Healthy eyes, and 5-neighborhoods are suitable for disease eyes with sharp changes in slope. If two or more candidate pixels have the same signal intensity, then the algorithm will refer to the extension direction of the previous step to make a selection. Figure 4c shows a schematic diagram of the extension of a single seed point. In the end, each seed point will form a path across the B-scan image.

(3) Boundary selection

The boundary path searched by a single seed point may have a certain deviation from the real boundary, but as the number of paths increases, there will be more and more paths superimposed on the real boundary. We use Eq. (3) to convert the number of superposed paths into probabilities. Now, the probability value at the real boundary is much higher than that at other locations.

3 $$ P\left( {i,j} \right) = \frac{1}{N} \cdot \mathop \sum \limits_{n = 1}^{N} B\left( {i,j,n} \right) \cdot 100 $$

**Figure 5:**
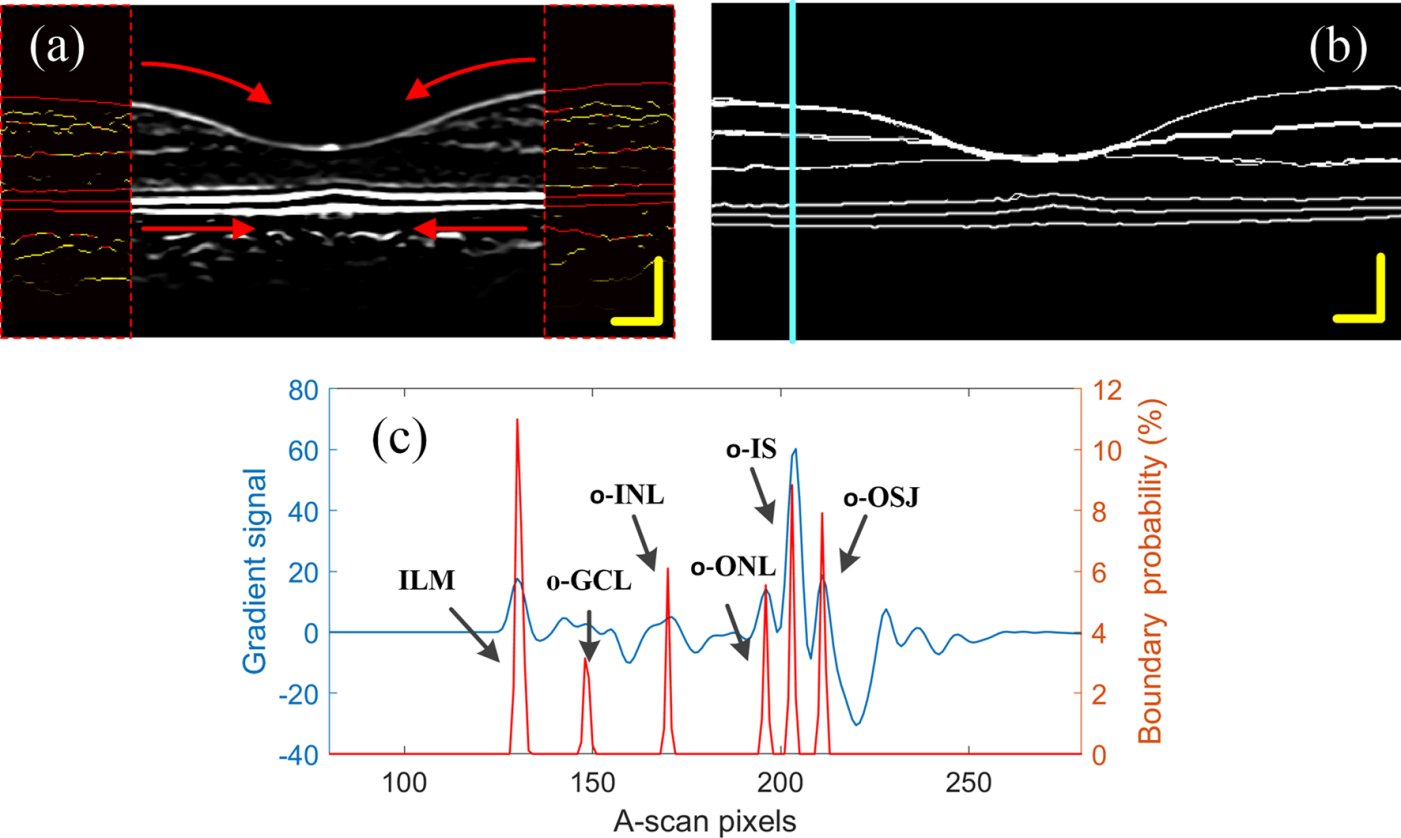
(**a**) The selection of the seed point area, (**b**) the obtained retinal boundary image, (**c**) the gradient signal and the boundary signal at the position of the blue solid line in (**b**).

Finally, the double threshold method in Canny operator was used to filter and connect the paths. Paths with a probability less than the low threshold are eliminated, and paths with a probability greater than the high threshold are retained. Then search for points greater than the low threshold at the breakpoints in the high-threshold image, until the edges of the entire image are closed. Finally, the retinal boundary image was obtained, as show in Fig. 5b.

If all the points in Fig. 3f are used as seed points for boundary search, it will be very time-consuming. In fact, according to the structural characteristics of the human eye's macula, it is only necessary to select the seed points on both sides of the image for calculation, that is, to search the boundary from both sides to the center, and an ideal result can be obtained. In this paper, 30-pixel-wide local regions on both sides of the image (Fig. 5a color regions) are selected, and the resulting retinal boundary image is shown in Fig. 5b. Figure 5c shows the gradient signal and boundary signal at the location shown by the solid blue line in Fig. 5b.

### Algorithm entire process and boundary identification

**Figure 6:**
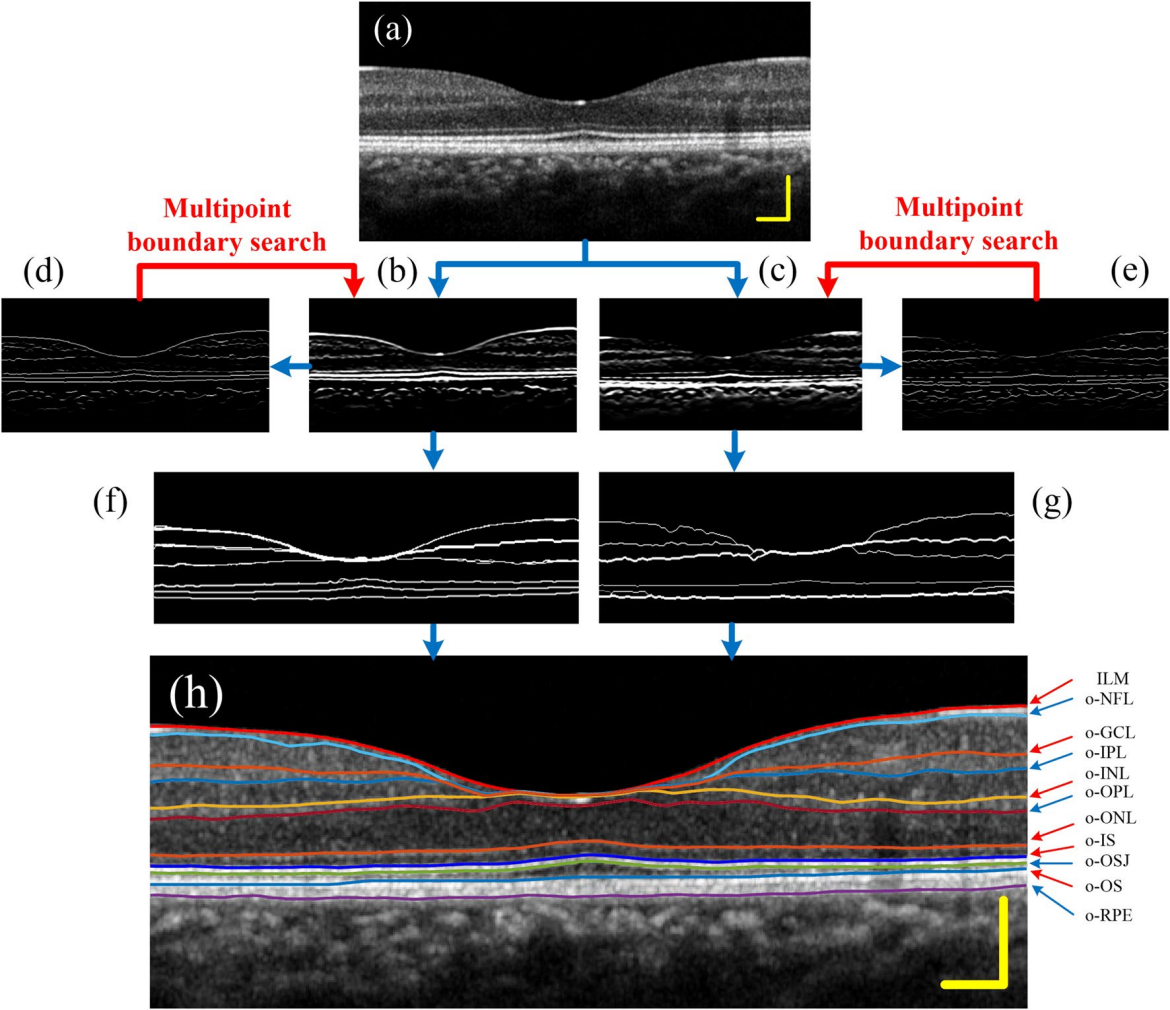
Segmentation results of the normal human retina. (**a**) Image after denoising. (**b**,**c**) Gradient images. (**d**,**e**) Peak point images. (**f**,**g**) Retinal boundary images. (**h**) Eleven retinal boundaries.

Figure 6 shows the segmentation results of a normal human retina. Figure 6a is the filtered image. Figure 6b,c are dark-to-bright and bright-to-dark boundary enhancement images, respectively. Figure 6d,e are peak point images after “non-maximum suppression” processing. Seed points were selected from the peak point images for path search, and the retinal boundary images were obtained, as shown in Fig. 6f,g. Finally, eleven boundary layers were identified from the two retinal boundary images in a specific order, as shown in Fig. 6h.

After the boundary image is obtained, eleven retinal boundaries are identified in a certain order. First, the dark to bright boundaries in Fig. 6f is detected. ILM is defined as the first highlighted reflection layer on each A-scan in the retinal image, which is most often well demarcated. Next, three outer layer boundaries from dark to light (o-ONL, o-IS, o-OS) are identified in sequence. Where, the o-IS layer generally has the highest brightness other than the ILM layer in Fig. 6d, so it can be identified in conjunction with Fig. 6d. In Fig. 6f, o-ONL and o-OS are located above and below the o-IS layer, respectively. ONL is a relatively obvious wide dark band. Above the o-ONL, the o-INL layer can be detected. Among the dark-to-bright retinal borders, only o-GCL has not yet been identified. In general, o-GCL is the most unclear layer in the image, so we put it at the end for recognition.

The next step is to determine the bright-to-dark boundaries. o-RPE has the highest brightness in Fig. 6e and can be identified in conjunction with Fig. 6e. o-OSJ is located between o-IS and o-OS, and the coordinates of o-OSJ can be restricted between o-IS and o-OS for identification. o-OPL and o-IPL are clearly visible and easy to identify. o-NFL is the first dark-to-bright interface under ILM. Finally, o-GCL is identified between o-NFL and o-IPL. All detected boundaries are smoothed using Gaussian filtering, and finally superimposed on the retinal structure map. The result is shown in Fig. 6h.

## Result

### Segmentation experiment results

We recruited 20 subjects (30 eyes), consisting of 10 healthy controls without ocular or systemic diseases, 10 patients with Mild AMD. Each healthy subject collected images of the left and right eyes, and AMD patients collected images of the diseased eye on one side. All subjects were recruited from the First Hospital of Qinhuangdao City. The study was conducted in accordance with the principles of the Declaration of Helsinki. This study also complies with the ethical guidelines for human medical research and the quality management norms for drug clinical trials. The research protocol was approved by the ethics committee of Qinhuangdao First Hospital. Informed consents were obtained from all participants.

The retinal OCT images used in this study were obtained from a Commercial Spectralis OCT System (based on Spectralis OCT; Heidelberg Engineering, Heidelberg, Germany). This device operates at 85 kHz A-scan rate, with a central wavelength of 870 nm and a bandwidth of 50 nm, and provides ~ 3.9-μm axial and 6-μm lateral resolution. The ocular light power exposure was within the American National Standards Institute safety limit. Each B-scan datum was composed of 512 A-scans.

**Figure 7:**
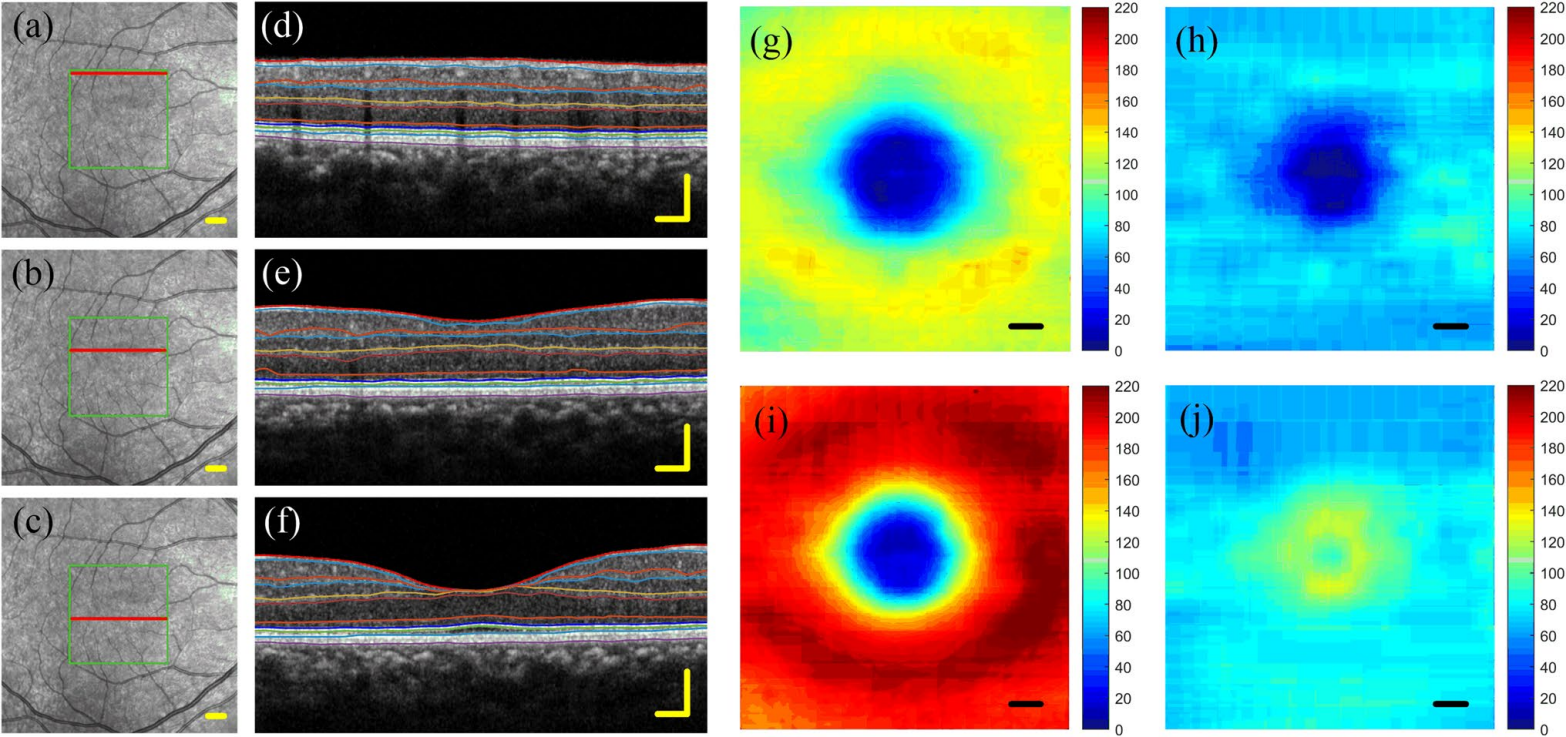
3D segmentation result of a healthy retina and the thickness maps of different layers. (**a**–**c**) The infrared fundus photography images of a healthy eye. The green box is the range of OCT imaging, and the red solid line points to the current B-scan position. (**d**–**f**) The retinal images and the layered results at the position shown by the solid red line. (**g**) The thickness map of superficial vascular complex (from ILM to o-IPL). (**h**) The thickness map of deep vascular complex (from o-IPL to o-OPL). (**i**) The thickness maps of macular inner retinal layers, which are the sum of (**g**,**h**). (**j**) The thickness map of macular outer retinal layers. The unit is μm.

Figure 7 shows the segmentation results of a healthy retina and the thickness map of different layers obtained by the algorithm proposed in this paper. Figure 7a–c are the infrared fundus photography images of healthy eyes. The green frame is the imaging range of the OCT, and the solid red line points to the current B-scan position. Figure 7d–f are the retinal images and the layered results at the position shown by the solid red line. Figure 7g shows the thickness map of the superficial vascular complex (from ILM to o-IPL). Figure 7h is the thickness map of the deep vascular complex (from o-IPL to o-OPL). Figure 7i is the thickness map of macular inner retinal layers, which is the sum of Fig. 7g,h. Figure 7j is the thickness map of macular outer retinal layers. The unit of thickness is μm.

**Figure 8:**
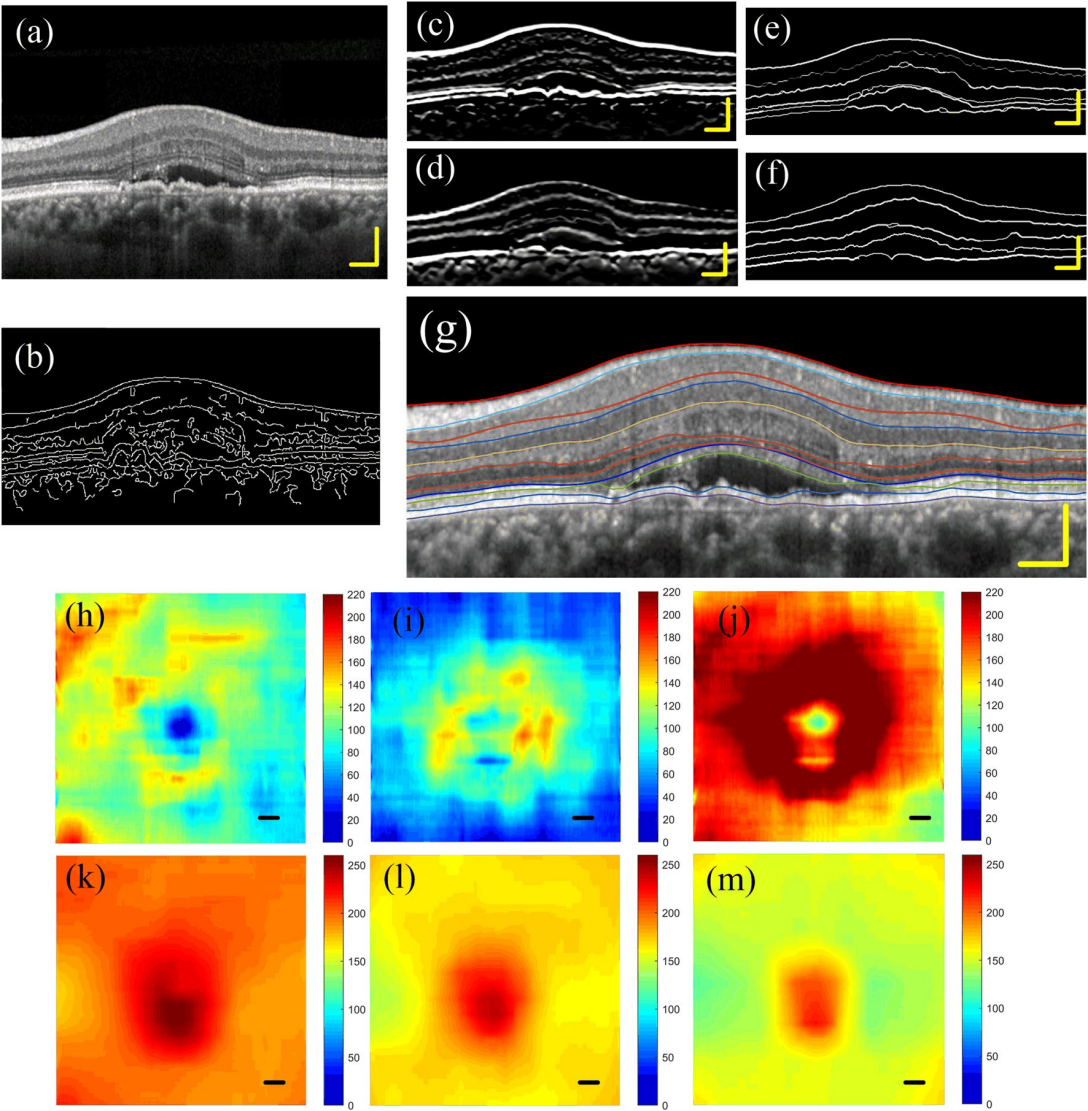
Segmentation result of an AMD eye. (**a**) A left retina of an AMD patient. (**b**) Boundary detection result obtained by using traditional canny operator. (**c**,**d**) dark-to-bright and bright-to-dark boundaries enhanced image. (**e**,**f**) retinal boundary images. (**g**) The segmentation results. (**h**–**j**) thickness maps of superficial vascular complex, deep vascular complex and macular inner retinal layers, respectively. (**k–m**) position maps of ILM, o-IPL and o-OPL.

This algorithm is also applicable to the stratification of mild AMD eyes. Figure 8a shows the left eye of a 60-year-old female patient who was diagnosed with wet age-related macular degeneration (wAMD). The best corrected visual acuity (BCVA) of the left eye was recorded as 20/40. As can be seen from Fig. 8a, the patient is accompanied by macular edema, subretinal fluid, neuroepithelial and drusenoid pigment epithelial detachments. Figure 8b is the boundary detection result obtained by using traditional canny operator. Figure 8c,d are dark-to-bright and bright-to-dark boundaries enhancement image, respectively. Figure 8e,f are retinal boundary images. Figure 8g is the segmentation results. Figure 8h–j are the thickness maps of superficial vascular complex, deep vascular complex and macular inner retinal layers, respectively. Figure 8k ~ (m) are the position maps of ILM, o-IPL and o-OPL. In normal eyes, the center of the macular area is lower than the surrounding area. However, for AMD eyes with macular edema, the macular fovea area is significantly higher than other surrounding areas. Therefore, we can see that the middle area in Fig. 8k–m is much higher than the surrounding area.

**Figure 9:**
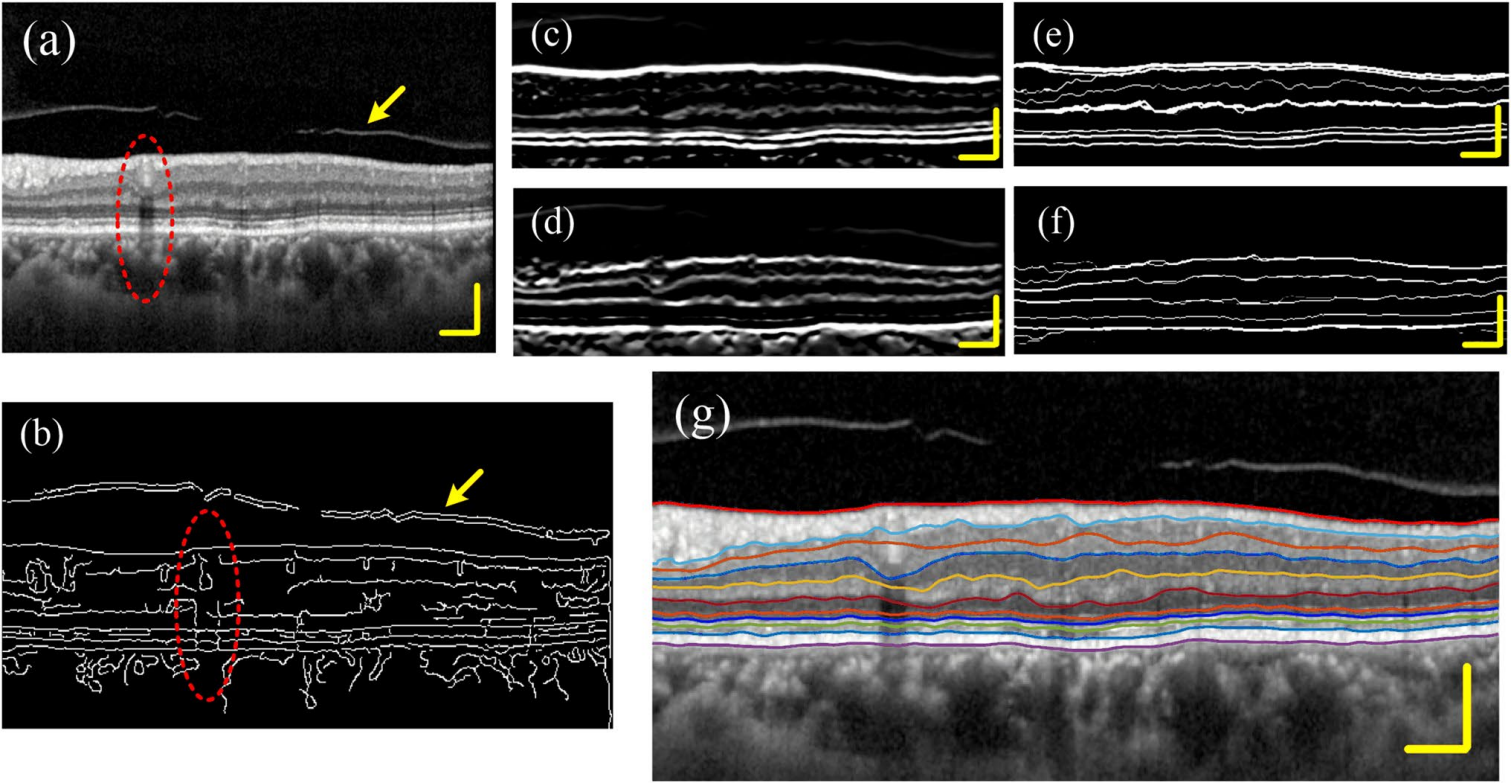
Segmentation results of disturbed image. (**a**) A retinal image that is disturbed by the posterior vitreous face. The red ellipse shows a shadow of blood vessel, and the yellow arrow shows the posterior vitreous face. (**b**) Boundary detection result obtained by using traditional canny operator. The axial boundary caused by blood vessel shadows can be seen. (**c**,**d**) Dark-to-bright and bright-to-dark boundaries enhanced images. (**e**,**f**) Retinal boundary images. (**g**) The segmentation results.

In the previous section, we show the segmentation results of some undisturbed retinal images. However, in practical applications, OCT retinal images are often subjected to some interference, such as posterior vitreous face, vascular artifacts and strong noise interference. Figure 9a is a retinal image with both posterior vitreous face (yellow arrow) and arterial artifacts (red arrow). Figure 9b is the boundary detection result obtained by using traditional canny operator. The axial boundary caused by the shadow of the blood vessel is very obvious; the boundary of the posterior vitreous face is also clearly displayed. Figure 9c–f is the corresponding boundary detection process using the proposed algorithm. Figure 9g is the final result. Compared with the traditional canny operator, the algorithm in this paper has a significant improvement in the detection ability of the retinal boundary.

**Figure 10:**
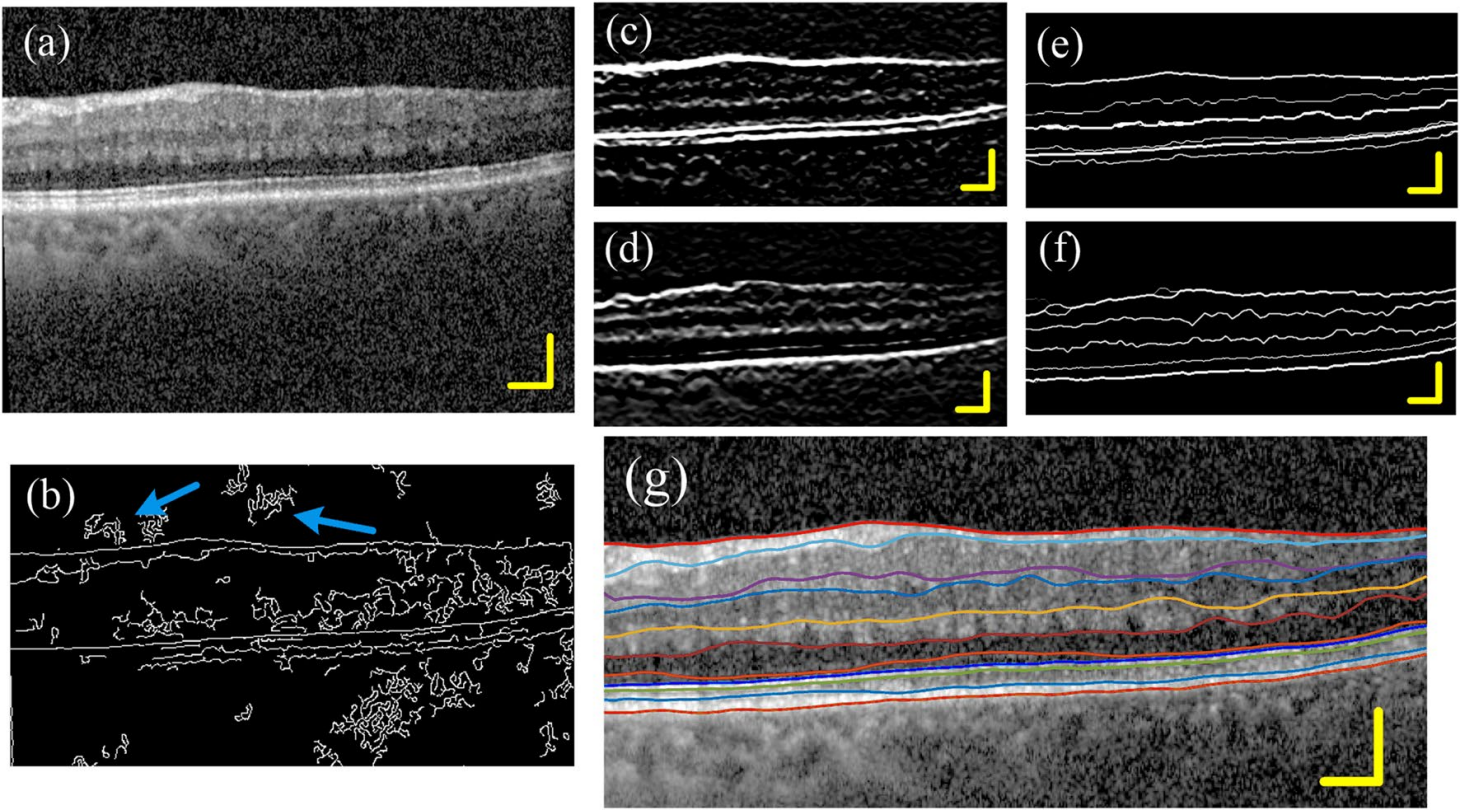
Segmentation results of noisy images. (**a**) A retinal image of noise interference. (**b**) Boundary detection result obtained by using traditional canny operator. The blue arrow indicates a false boundary caused by noise. (**c**,**d**) Dark-to-light and light-to-dark boundaries enhanced image. (**e**,**f**) Retinal border images. (**g**) The segmentation results.

The quality of OCT images often deteriorates due to the subject's eye jitter, opacity of the refractive interstitium, or improper operation. It may also be due to the strong speckle noise superimposed on structural images, which reduces the image contrast near the layer boundaries. Figure 10 shows the segmentation results of a set of noisy images. It can be seen that the traditional canny operator is very sensitive to noise interference, and the algorithm in this paper is hardly affected.

### Quantitative evaluation

The quantitative evaluation is achieved by comparing the boundary positioning difference between the proposed automated method and the manual segmentation method. Among them, manual segmentation method is implemented by four experienced ophthalmologist using Photoshop software (Adobe Systems Inc.). They draw 11 retinal boundaries on the B-scan retinal images. The difference in the axial location of the boundary obtained by the two methods was quantified using the mean unsigned positioning error (MUE)^43^. For a particular boundary, the MUE was defined by

4 $$ {\text{MUE}}\left( {{\text{L}}_{1} ,{\text{L}}_{2} } \right) = \frac{1}{N} \cdot \mathop \sum \limits_{j = 1}^{N} \left| {L_{1} \left( j \right) - L_{2} \left( j \right)} \right| $$

where L1 and L2 are the results of automatic and manual segmentation of a certain retinal layer, respectively. N is the number of A-scans included in the B-scan. The eyes of 20 healthy subjects and 10 eyes of AMD patients were compared and analyzed. The quantitative comparison results are shown in Tables 1 and 2.

**Table 1:** Quantitative comparison of boundary positioning between automated and manual segmentation in healthy subjects’ eyes.

Retinal layer	Healthy subjects MUE (mean ± SD)			
	Reader 1	Reader 2	Reader 3	Reader 4
ILM	1.42 ± 1.08	1.66 ± 0.74	1.51 ± 0.57	1.83 ± 0.42
o-NFL	3.06 ± 1.75	3.30 ± 1.25	3.60 ± 1.87	4.32 ± 1.59
o-GCL	4.73 ± 2.71	4.52 ± 2.94	4.96 ± 2.76	7.68 ± 2.34
o-IPL	3.18 ± 1.42	3.45 ± 0.89	3.24 ± 0.95	3.55 ± 1.87
o-INL	2.87 ± 1.73	4.04 ± 1.96	2.59 ± 0.77	3.22 ± 1.71
o-OPL	3.18 ± 1.92	4.28 ± 1.09	3.01 ± 1.27	3.36 ± 2.37
o-ONL	3.02 ± 1.75	2.95 ± 0.84	3.08 ± 0.96	1.91 ± 1.04
o-IS	3.49 ± 1.86	2.63 ± 0.73	3.39 ± 0.97	2.16 ± 1.75
o-OSJ	3.73 ± 1.61	2.95 ± 0.69	3.67 ± 0.57	3.35 ± 2.21
o-OS	4.39 ± 1.87	3.71 ± 1.15	4.69 ± 1.90	3.75 ± 3.37
o-RPE	4.90 ± 2.27	3.17 ± 1.29	5.19 ± 0.94	3.73 ± 1.19

*MUE* mean unsigned positioning error, the unit is μm.

**Table 2:** Quantitative comparison of boundary positioning between automated and manual segmentation in AMD patients’ eyes.

Retinal layer	AMD patients MUE (mean ± SD)			
	Reader 1	Reader 2	Reader 3	Reader 4
ILM	2.14 ± 0.88	2.75 ± 1.23	2.71 ± 1.45	2.77 ± 2.10
o-NFL	3.61 ± 1.10	4.17 ± 2.67	5.76 ± 2.07	4.84 ± 7.14
o-GCL	5.82 ± 1.54	7.60 ± 2.17	7.10 ± 1.80	6.08 ± 6.53
o-IPL	3.49 ± 0.74	4.57 ± 1.16	5.23 ± 1.66	2.73 ± 3.55
o-INL	3.14 ± 0.82	4.19 ± 1.84	4.65 ± 1.52	3.57 ± 1.91
o-OPL	5.09 ± 2.17	6.04 ± 2.86	8.36 ± 3.99	10.1 ± 4.02
o-ONL	4.74 ± 1.53	5.32 ± 1.91	8.18 ± 2.54	5.11 ± 3.56
o-IS	3.96 ± 1.04	4.08 ± 1.74	6.15 ± 2.18	3.77 ± 1.69
o-OSJ	7.17 ± 3.02	4.56 ± 1.25	8.01 ± 5.9	4.69 ± 3.63
o-OS	9.67 ± 3.37	6.13 ± 2.79	10.8 ± 5.9	7.53 ± 6.71
o-RPE	8.11 ± 6.23	6.35 ± 4.59	7.81 ± 6.59	7.86 ± 7.19

*MUE* mean unsigned positioning error, the unit is μm.

**Figure 11:**
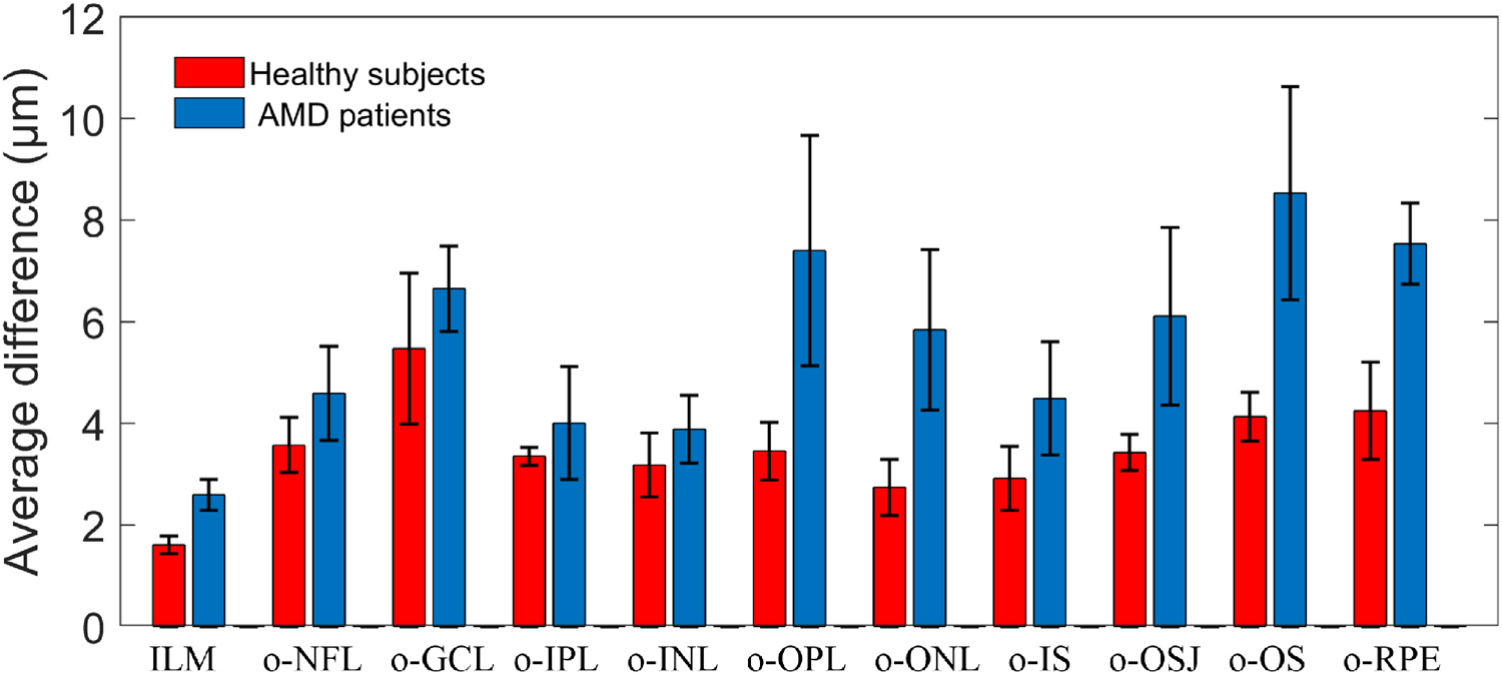
Average difference between automated and manual segmentation of healthy subjects and AMD patients.

We once again averaged the 4 columns of data (mean values) in Tables 1 and 2 to obtain the histogram as shown in Fig. 11, which can provide a reference for evaluating the accuracy of the proposed algorithm for retinal segmentation in healthy subjects and AMD patients. The red box in the Fig. 11 represents the average difference of healthy subjects (the mean of the 4 columns of data in Table 1), and the blue box represents the average difference of AMD patients (the mean of the 4 columns of data in Table 2), the error bars represent standard deviation.

It can be seen from Fig. 11 that the difference between the automatic and manual segmentation results of the o-GCL layer of healthy subjects is larger than that of other layers, with an average difference of about 6 microns (less than 2 pixels). The difference in other layers is about 2–4 microns. The average difference between o-OPL and o-RPE in AMD patients was significantly larger than that in healthy subjects. The maximum difference is about 10 microns. The increase in the average difference can only explain the difficulty of identifying the boundary and the uncertainty of the segmentation result, but it cannot explain which method is absolutely accurate, because manual segmentation is not a gold standard either. Therefore, we need to perform further qualitative assessment.

### Qualitative evaluation

The qualitative evaluation is achieved by inviting professional readers to score the automatic segmentation results, with a score ranging from 1 to 4 points. A score of 1 indicates a Perfect or close to perfect boundary location; a score of 2 indicates a Good boundary location, which may require minor corrections. A score of 3 indicate major problems with boundary location determination and a score of 4 for failing to produce any result. Each retinal layer is scored separately and the average and standard deviation are calculated to highlight the algorithm's ability to recognize different retinal layers. All readers who received the invitation, whether for quantitative or qualitative evaluation, were senior ophthalmologists from the First Hospital of Qinhuangdao City. The qualitative evaluation results are shown in Tables 3 and 4.

**Table 3:** Qualitative evaluation in Healthy subjects.

Retinal layer	Healthy subjects score (mean ± SD)		
	Reader 1	Reader 2	Reader 3
ILM	1.0 ± 0	1.1 ± 0.31	1.1 ± 0.31
o-NFL	1.2 ± 0.41	1.1 ± 0.31	1.2 ± 0.41
o-GCL	1.6 ± 0.88	1.8 ± 0.42	1.2 ± 0.41
o-IPL	1.1 ± 0.31	1.7 ± 0.48	1.1 ± 0.31
o-INL	1.2 ± 0.41	1.8 ± 0.42	1.3 ± 0.47
o-OPL	1.3 ± 0.47	1.9 ± 0.32	2.2 ± 0.42
o-ONL	1.1 ± 0.31	1.4 ± 0.52	1.0 ± 0
o-IS	1.1 ± 0.31	1.3 ± 0.47	1.0 ± 0
o-OSJ	1.1 ± 0.31	1.1 ± 0.31	1.5 ± 0.53
o-OS	1.2 ± 0.41	1.1 ± 0.31	2.3 ± 0.48
o-RPE	1.1 ± 0.31	1.2 ± 0.41	1.4 ± 0.52

**Table 4:** qualitative evaluation in AMD patients.

Retinal layer	AMD patients score (mean ± SD)		
	Reader 1	Reader 2	Reader 3
ILM	1.1 ± 0.31	1.1 ± 0.31	1.1 ± 0.31
o-NFL	1.3 ± 0.47	2.2 ± 0.42	1.1 ± 0.31
o-GCL	1.7 ± 0.48	2.8 ± 0.42	1.5 ± 0.53
o-IPL	1.1 ± 0.31	2.2 ± 0.42	1.2 ± 0.41
o-INL	1.2 ± 0.41	2.4 ± 0.52	1.2 ± 0.41
o-OPL	1.4 ± 0.52	2.2 ± 0.42	2.0 ± 0.47
o-ONL	1.1 ± 0.31	1.8 ± 0.42	1.9 ± 0.32
o-IS	1.1 ± 0.31	1.5 ± 0.53	1.5 ± 0.53
o-OSJ	1.3 ± 0.47	1.2 ± 0.41	1.8 ± 0.42
o-OS	2.2 ± 0.42	1.1 ± 0.31	1.3 ± 0.47
o-RPE	1.2 ± 0.41	1.1 ± 0.31	1.2 ± 0.41

**Figure 12:**
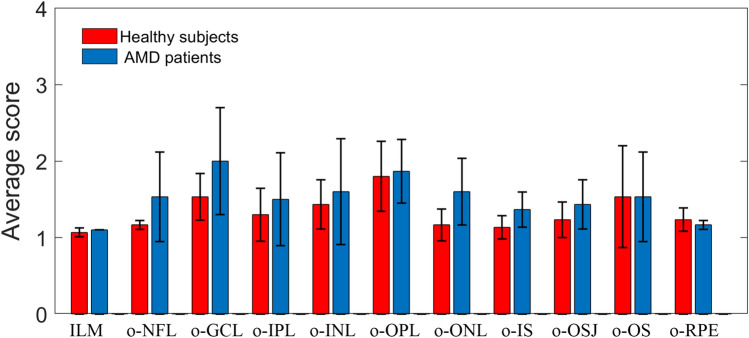
Average scores of healthy subjects and AMD patients.

The 3 columns of data (average values) in Tables 3 and 4 are averaged again to obtain a histogram as shown in Fig. 12. The red columns represent the average score of healthy subjects (the average of the 3 columns of data in Table 3), and the blue columns represent the average score of AMD patients (the average of the 3 columns of data in Table 4), the error bars indicate standard deviation.

In Fig. 12, the average scores of AMD patients and healthy subjects are very close. It shows that the accuracy of the segmentation results obtained by the proposed algorithm is very high, and some differences are within the acceptable or adjustable range.

### Comparison with a state-of-the-art method

The proposed method was compared with a state-of-the-art retinal segmentation method^43^. We used the same public data set as the literature^43^, which collected by Farisu et al.^46^, can be downloaded online. The data set consists of a total of 384 SD-OCT macular scans. Of these, 115 scans were from healthy subjects, and the remaining 269 scans were from patients with non-exudative AMD of varying severity. Subjects' ages and the corresponding manually corrected reference standards for three retinal layer boundaries are also included. These reference segmentation boundaries were obtained using their proposed segmentation algorithm^39^ and later corrected manually by experienced graders (certified by the Duke Advanced Research in Spectral Domain OCT Imaging laboratory): The SD-OCT scans were acquired using Bioptigen, Inc. (Research Triangle Park, NC) imaging systems at four different clinical sites. Each SD-OCT cube in the second data set consists of 1000 (horizontal) × 100 (vertical) × 512 (axial) voxels, covering dimensions of approximately 6.7 (horizontal) × 6.7 (vertical) × 1.6 (axial) mm. In the second data set, the voxel dimensions in the horizontal, vertical, and axial directions were approximately 6.7, 67, and 3.1-μm respectively.

**Figure 13:**
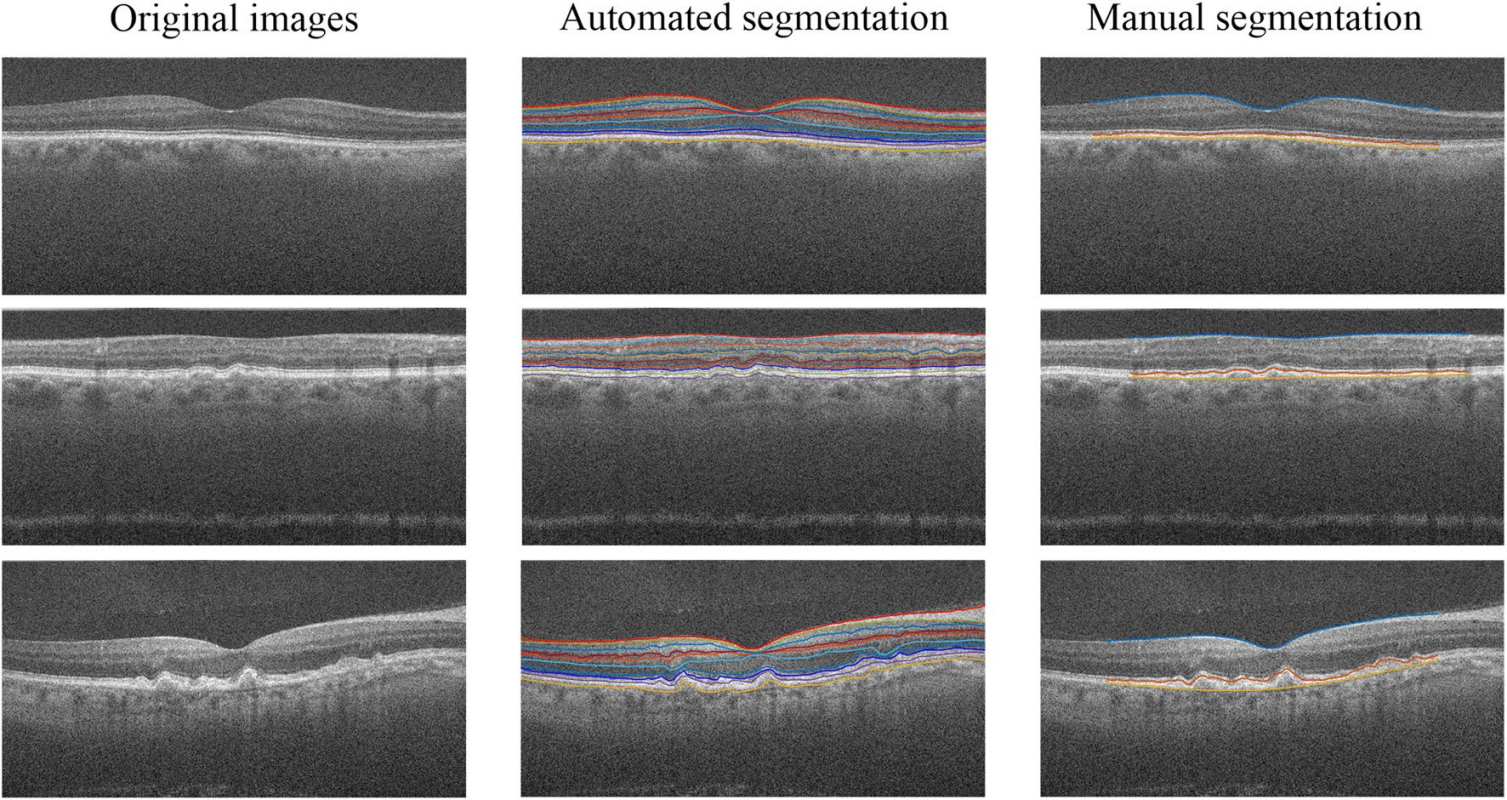
The segmentation result of public data set obtained by the proposed algorithm. The first row is b-Scan images of healthy subjects, and the second and third rows are B-scan images of AMD patients.

Figure 13 shows the segmentation results of the public data set obtained by the algorithm in this paper. The first row shows B-scan images of a typical healthy subject, and the second and third rows show B-scan images of AMD patients. The first column is the original image, and the second column is the automatic segmentation result obtained by using the algorithm in this paper. The third column is manually corrected reference standards boundaries of the ILM, O-OS and O-RPE, which is used to test the accuracy of different methods.

Table 5 shows the comparison results of the algorithm in this paper and the algorithm in literature^43^. Regarding the identification of the ILM layer, the accuracy of the proposed method is significantly higher than that of the method in the literature^43^, both in healthy subjects and AMD patients. For the o-OS layer, the recognition accuracy of the proposed method is similar to that of healthy people in the literature^43^, and the recognition accuracy of AMD patients is slightly lower. The reason may be that the algorithm in this paper is weak in recognizing violent fluctuating boundaries, and needs subsequent improvement.

**Table 5:** Comparison between the proposed method and that in literature^43^.

Retinal layer	Proposed method MUE			IS-WM^43^ MUE		
	Healthy subjects	AMD patients	All scans	Healthy subjects	AMD patients	All scans
ILM	2.36 ± 1.15	2.88 ± 1.92	2.77 ± 1.75	3.47 ± 4.15	6.43 ± 11.30	5.51 ± 9.76
o-OS	5.35 ± 3.55	8.39 ± 6.81	7.48 ± 6.15	5.41 ± 3.77	7.07 ± 5.66	6.56 ± 5.20
Average	**3.86 ± 2.79**	**5.64 ± 5.72**	**5.13 ± 5.09**	4.44 ± 4.07	6.75 ± 8.93	6.04 ± 7.83

*MUE* mean unsigned positioning error, the unit is μm.

## Discussion

Retinal layering algorithms have been developed for many years. The existing methods have one or more of the following disadvantages: they distinguish only the most prominent layers, they do not exhibit robustness under noisy and changing conditions, the algorithm is very time-consuming or the algorithm design is very complicated. Most importantly, most algorithms require more or less human intervention, such as setting the initial position, setting the search interval, and setting different algorithm parameters or thresholds for different data sets. These excessive human interventions reduce the objectivity of the algorithm and hinder the use and development by medical staffs. Therefore, the development of an automatic retinal layering algorithm with high accuracy and strong anti-interference ability is of great significance for the diagnosis and research of ophthalmological diseases.

This paper proposed a retinal layer segmentation algorithm based on improved Canny operator. The conventional canny operator consists of four steps: 1. Image denoising; 2. Gradient calculation; 3. Non-maximum suppression; 4. Using dual threshold method to select and connect edges. Compared with the traditional canny operator, the method in this paper has the following improvements:

1. The traditional canny operator is susceptible to the shadow of the blood vessel, resulting in an edge perpendicular to the retinal layer. This is because the canny operator uses the non-directional Sobel operator as the convolution kernel function to obtain the image gradient value and gradient direction. It can monitor not only the horizontal retinal boundary layer, but also the vertical vascular shadow boundary. This paper uses the axial gradient template to calculate the image gradient, and only horizontal boundary is highlighted. Additionally, the algorithm uses the method of multiplying gradient images and the large-scale smooth images to enhance the boundaries of each retina layer, greatly suppressing noise and interference outside the retina.
2. Affected by blood vessel shadow or image quality, the boundary detected by the traditional Canny operator is often discontinuous. In this paper, a multi-point boundary search step is added on the basis of the traditional canny operator. Several peak points were selected from the non-maximum suppression image as seed points, and then were extended to both sides in the gradient enhanced image using a boundary search method to form a path. When encountering the shadow of the blood vessel, the algorithm will continue the search direction of the previous step and continue to extend forward. In this way, the interference of blood vessel shadows is well avoided.
3. The traditional canny operator is sensitive to noise or interference, and is prone to misdetection or missed detection. In this paper, a certain number of seed points were used to search the boundary together and the boundaries formed by all the seed points were superimposed on one image. The number of times the boundary is overlapped is converted into the boundary probability. In general, the real boundaries are bound to have higher probabilities. Such boundary detection method that relies on the "group effect" can accurately detect the retinal boundary, even in the case of noise interference, the accurate boundary position can be found with a high probability. The use of the dual-threshold method further guarantees the accuracy and completeness of the boundary. The main advantage of the algorithm is that it almost does not require any manual involvement (including A-lines alignment, manual initialization, parameter adjustment or search space restriction, etc.), and its accuracy and stability are also very satisfactory.

In this paper, various types of retinal images are used for testing, and ideal segmentation results were obtained. This shows that the algorithm has strong accuracy and robustness in dealing with the interference of posterior vitreous face, blood vessel shadow, noise and lesions. The quantitative and qualitative evaluation results also fully confirmed this point. From the quantitative evaluation results, the average difference between the automatic segmentation algorithm and the manual segmentation algorithm is: 2–6 microns (1–2 pixels) for healthy subjects, 3–10 microns (1–3 pixels) for patients with mild AMD. From the qualitative evaluation results, the proportion of scores of 1 or 2 is 98% of healthy subjects and 94% of AMD patients.

The method described in this paper was implemented using MATLAB (The MathWorks, Inc.) M-file code. The program runs on a personal computer (64 bit OS, Intel Core i7 CPU at 3.6 GHz, and 8 GB RAM) and took about 124 s to complete the whole 3-D image volume (480 × 512 × 300) pixels detection of eleven layer boundaries. The average processing time for each B-Scan is 242 ms. If a more efficient language was used, for example C+   programming language, the program can perform with dramatically reduced processing time. If the segmentation process is performed once every 4 B-scans, and then the interpolation method is used to fill the gaps, then only 128 retinal segmentations need to be performed for 512 B-scans. The time consumed will be reduced to 1/4 of the original. The processing speed of the proposed method is much higher than that of the method in reference^43^ and slightly lower than that of the method in reference^47^ (the fastest retinal segmentation method to date). However, the number of retinal layers identified by the proposed method (11 layers) is greater than that by the method in reference (8 layers)^47^.

The algorithm currently does not add excessive error correction processing based on prior information. Therefore, there is still a lot of room for improvement in algorithm performance. It is worth noting that the method in this paper has a very high accuracy rate for retinal segmentation for patients with mild AMD, but for severe AMD patients, the recognition accuracy will decrease due to the increase in subretinal fluid and severe retinal structural deformation. This algorithm can be used alone or in combination with other methods as an initial boundary detection algorithm to improve the ability to detect AMD retinal boundaries. The proposed technique can also be extended to segment other hierarchical structures.

In summary, the retinal segmentation algorithm based on the improved Canny operator proposed in this paper is a robust and automatic algorithm that can distinguish eleven retinal boundaries without human intervention; the segmentation results have high accuracy and stability. The algorithm is easy to understand and improve, and has the potential to become a powerful tool for analyzing and diagnosing eye diseases.

## Supplementary Information

Supplementary Information 1.

Supplementary Information 2.

Supplementary Information 3.

Supplementary Information 4.

Supplementary Information 5.

Supplementary Information 6.
