# Supplementary figures and images for: Automated retinal boundary segmentation of optical coherence tomography images using an improved Canny operator
Source: Sci Rep. 2022 Jan 26;12:1412. doi: 10.1038/s41598-022-05550-y (PMC8791938; doi:10.1038/s41598-022-05550-y)

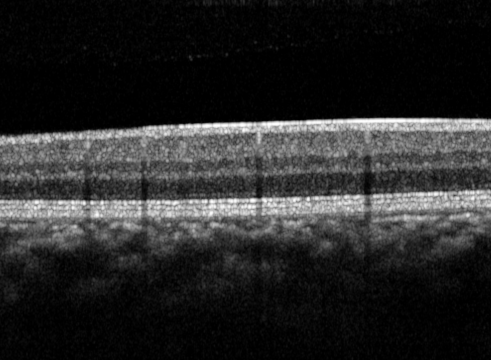

Supplement: Supplementary file 1 — Supplementary Information 1. [file 41598_2022_5550_MOESM1_ESM.tif]

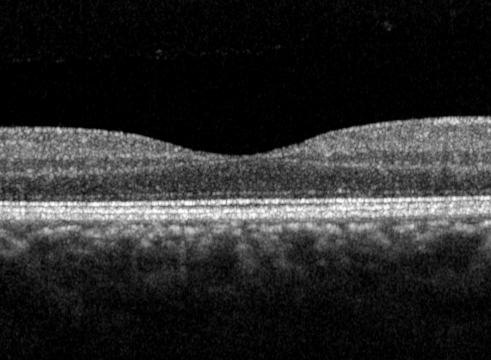

Supplement: Supplementary file 2 — Supplementary Information 2. [file 41598_2022_5550_MOESM2_ESM.tif]

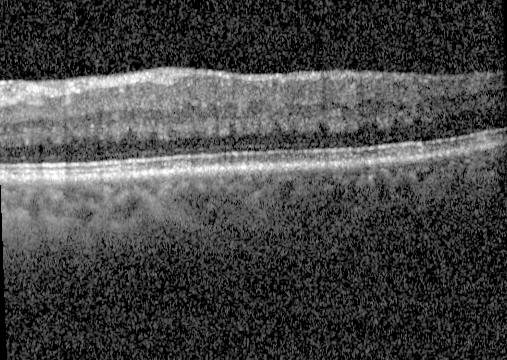

Supplement: Supplementary file 3 — Supplementary Information 3. [file 41598_2022_5550_MOESM3_ESM.tif]

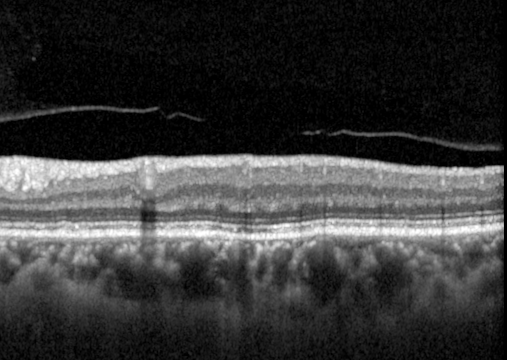

Supplement: Supplementary file 4 — Supplementary Information 4. [file 41598_2022_5550_MOESM4_ESM.tif]

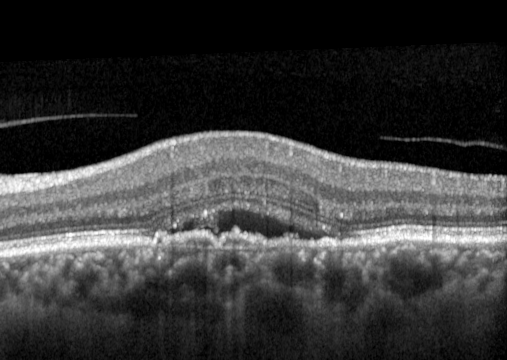

Supplement: Supplementary file 5 — Supplementary Information 5. [file 41598_2022_5550_MOESM5_ESM.tif]
