# Supplementary material for: Automated retinal boundary segmentation of optical coherence tomography images using an improved Canny operator
Source: Sci Rep. 2022 Jan 26;12:1412. doi: 10.1038/s41598-022-05550-y (PMC8791938; doi:10.1038/s41598-022-05550-y)
Supplement: Supplementary file 6 — Supplementary Information 6. [file 41598_2022_5550_MOESM6_ESM.docx]

The codes we used for our algorithm can be found in <https://github.com/Luna1985/a-series-of-OCT-code.git>, and the related figures are shown in Fig S1- Fig S5.

Fig S1 Macular marginal retina of healthy group

Fig S2 Macular center retina of healthy group

Fig S3 Retina with noise interference

Fig S4 Retina with both posterior vitreous face and arterial artifacts

Fig S5 Retina of a AMD patient
